# Bone graft migration into the spinal canal after endoscopic lumbar interbody fusion: a rare postoperative complication

**DOI:** 10.1055/s-0044-1793911

**Published:** 2024-12-07

**Authors:** Isaac C. Slagel, Mohammed Sabawi, Leonardo Furtado Freitas

**Affiliations:** 1 University of Iowa Health Care, Carver College of Medicine, Iowa City IA, United States.; 2 University of Iowa Hospitals and Clinics, Division of Neuroradiology, Department of Radiology, Iowa City IA, United States.

We herein report the case of a 57-year-old man who presented with sudden sharp pain radiating down his left side, the day after being subjected to minimally invasive spinal transforaminal lumbar interbody fusion (MIS TLIF) for L5–S1 spondylolisthesis. The procedure also included anterior fusion using demineralized bone fiber (DBF) allograft. Images of the spine revealed the allograft material's extrusion into the left L5–S1 epidural space and neuroforamen ( Figures 1 , 2 , 3 , 4 ). The patient underwent additional surgery to remove the migrated graft and alleviate nerve root compression.

**Figure 1:**
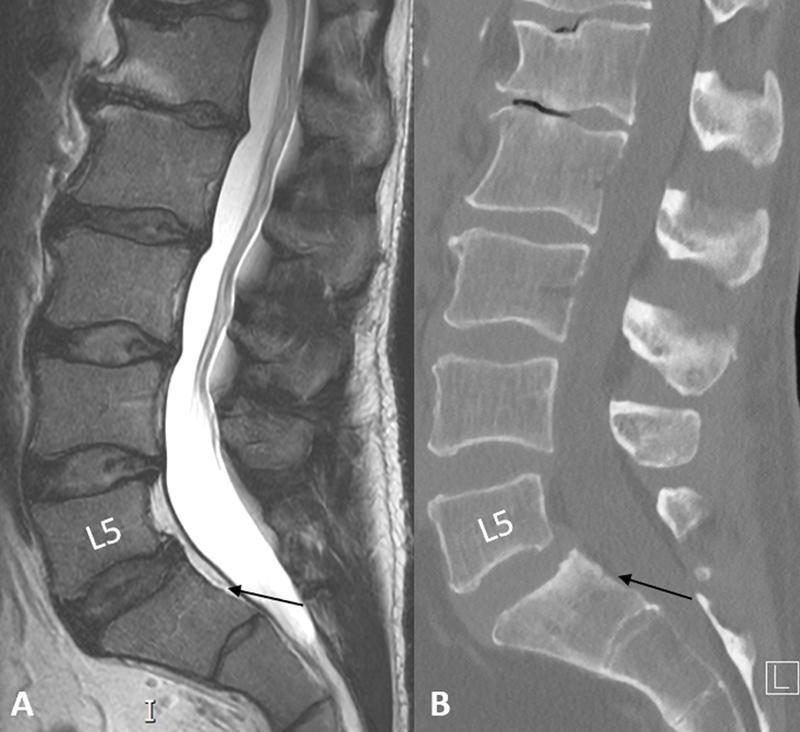
Preoperative ( **A** ) sagittal magnetic resonance imaging T2-weighted images and ( **B** ) Computed tomography bone window. Multilevel lumbar degenerative spondylosis, L5 vertebral body hypoplasia, and grade 1 anterolisthesis of L5 over S1 due to bilateral pars articularis defect (not shown). There was no anterior epidural tissue at the L5–S1 level (black arrows).

**Figure 2:**
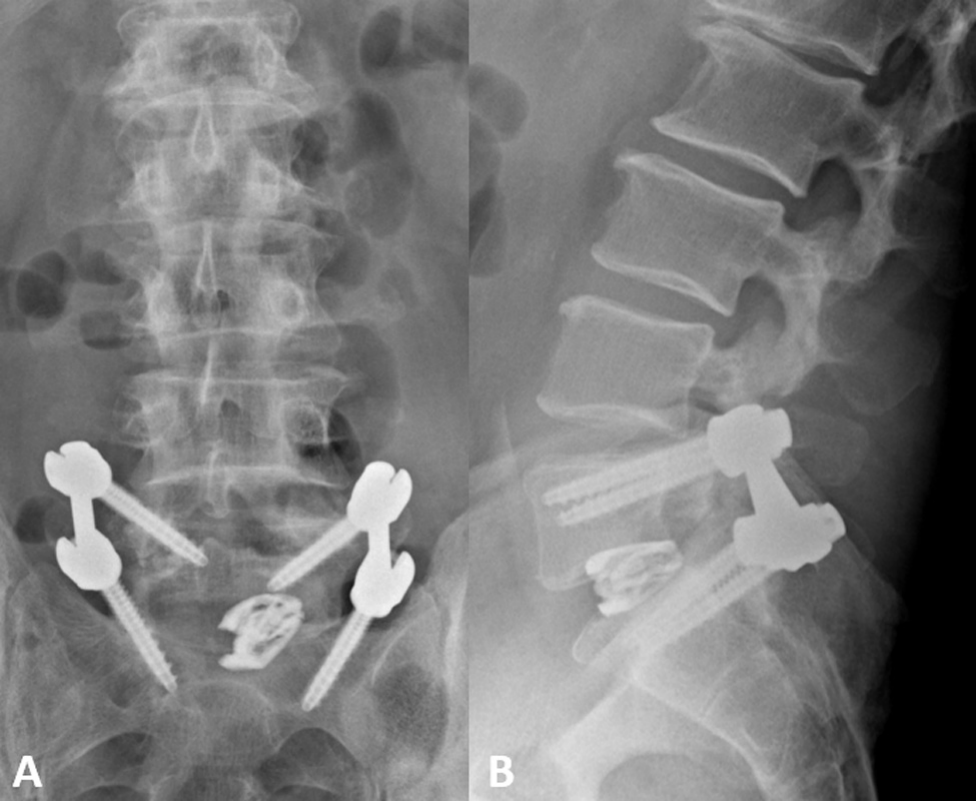
Postoperative lumbar spine X-ray 1 day after the procedure. ( **A** ) anteroposterior and ( **B** ) lateral views. The L5–S1 transforaminal lumbar interbody fusion with interbody spacer. No overt signs of hardware complication. In particular, there was no detectable component in the anterior epidural tissue at the L5–S1 level.

**Figure 3:**
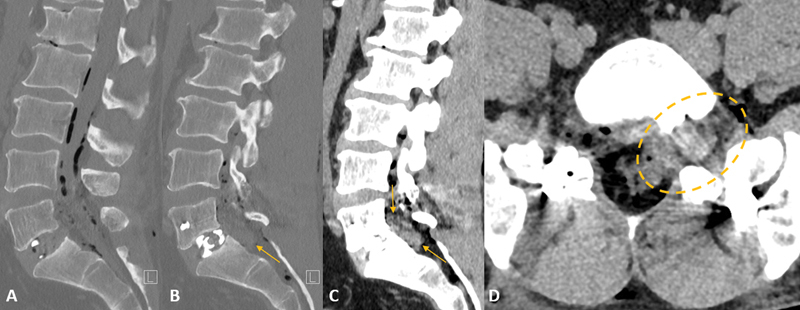
Postoperative lumbar spine computed tomography after 1-day. ( **A** ) Sagittal midline and ( **B** ) left paramedian bone window, and ( **C** ) sagittal left paramedian and ( **D** ) axial soft-tissue window. The normal placed L5–S1 transforaminal lumbar interbody fusion with interbody spacer. Soft-tissue density material in the L5–S1 anterior left epidural space and left neuroforamen (yellow arrows and dashed circle). There were residual air foci within the spinal canal.

**Figure 4:**
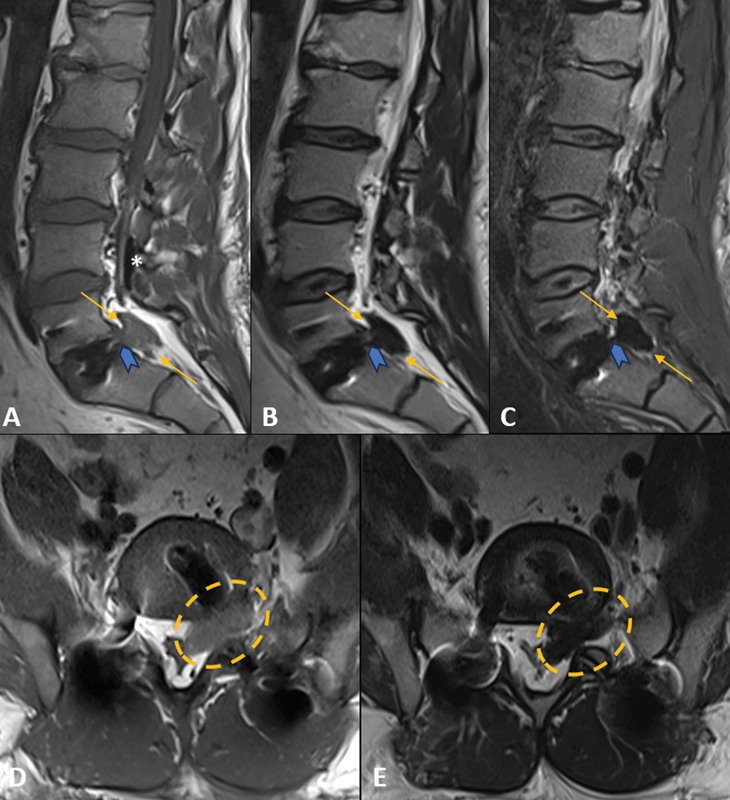
Postoperative lumbar spine magnetic resonance imaging after 1-day. ( **A** ) Left paramedian sagittal T1-weighted, ( **B** ) T2-weighted **,** and ( **C** ) Short-tau inversion recovery (STIR)-weighted images. ( **D** ) Axial T1- and ( **E** ) T2-weighted images. Normal placed L5–S1 transforaminal lumbar interbody fusion with interbody spacer. Greater conspicuity of the T1 isointense and T2/STIR markedly hypointense amorphous material in the L5–S1 level, occupying the anterior left epidural space and left neuroforamen (yellow arrows and dashed circle), with abutment of the left L5 exiting and S1 descending nerve roots. There was a well-defined pedicle (blue arrowheads) at the corresponding intervertebral disc. Redemonstrated residual air foci within the spinal canal, mainly in the posterior L4 epidural space (white asterisk).

This case highlights a rare complication in MIS TLIF, 1 2 3 possibly derived from insecure placement, intraoperative tissue damage, segmental instability, and/or excessive curettage of disc material.

## References

[1] Xie Y Zhou Q Wang Y Feng C Fan X Yu Y Postoperative bone graft migration into the thecal sac and shifting down to the lower level after an endoscopic lumbar interbody fusion: a case report BMC Musculoskelet Disord 2023 24 01 143 10.1186/s12891-023-06247-7PMID: 36823613; PMCID: PMC9948321 36823613 PMC9948321

[2] He H Xu J Posterior migration of bone-graft particles to the spinal canal after lumbar fusion: A case report Asian J Surg 2023 46 04 1842 1843 10.1016/j.asjsur.2022.10.063PMID: 36328845 36328845

[3] Rajadurai J Lovric V Mobbs R J Choy W J Walsh W R The use of demineralised bone fibres (DBF) in conjunction with supercritical carbon dioxide (SCCO _2_ ) treated allograft in anterior lumbar interbody fusion (ALIF) J Spine Surg 2019 5 04 589 595 10.21037/jss.2019.10.04PMID: 32043009; PMCID: PMC6989944 32043009 PMC6989944

